# Bubble-Enhanced Mixing Induced by Standing Surface Acoustic Waves (SSAWs) in Microchannel

**DOI:** 10.3390/mi13081337

**Published:** 2022-08-18

**Authors:** Jingjing Zhang, Tengfei Zheng, Lin Tang, Hui Qi, Xiaoyu Wu, Linlong Zhu

**Affiliations:** 1 School of Mechatronics Engineering, Xi’an Technological University, Xi’an 710021, China; 2 State Key Laboratory for Manufacturing Systems Engineering, Xi’an Jiaotong University, Xi’an 710049, China; 3 Shaanxi Key Laboratory of Intelligent Robots, Xi’an Jiaotong University, Xi’an 710049, China

**Keywords:** surface acoustic wave, acoustic micromixing, bubbles, acoustic cavitation

## Abstract

BAW-based micromixers usually achieve mixing enhancement with acoustic-induced bubbles, while SAW-based micromixers usually enhance mixing efficiency by varying the configuration of IDTs and microchannels. In this paper, bubble-enhanced acoustic mixing induced by standing surface acoustic waves (SSAWs) in a microchannel is proposed and experimentally demonstrated. Significant enhancement in the mixing efficiency was achieved after the bubbles were stimulated in our acoustofluidic microdevice. With an applied voltage of 5 V, 50 times amplified, the proposed mixing microdevice could achieve 90.8% mixing efficiency within 60 s at a flow rate of 240 μL/h. The bubbles were generated from acoustic cavitation assisted by the temperature increase resulting from the viscous absorption of acoustic energy. Our results also suggest that a temperature increase is harmful to microfluidic devices and temperature monitoring. Regulation is essential, especially in chemical and biological applications.

## 1. Introduction

With the advantages of a shorter reaction time, lower analysis costs, and portability, microfluidic systems have been widely used in a range of chemical, biological, and biomedical applications [1,2,3,4]. A micromixer is among the important components in microfluidic systems [5]. However, the small size of the microchannel and the increased interaction between the fluid and the surrounding channel walls restrict the Reynolds number to a small quantity; therefore, only laminar flow processes are allowed [6]. The domination of viscous effects at small scales leads to a low mixing efficiency in microfluidic systems. To achieve rapid and efficient micromixing between viscous fluids, various passive and active actuating approaches have been developed [7,8].

Passive micromixers increase the interfacial disturbance by well-defined geometry to produce chaotic or laminar mixing [9,10,11,12,13]. The dependence of passive micromixers on elaborate microchannel designs complicates the fabrication processes. On the contrary, active micromixers introduce external energy actuation, such as electro-kinetic, electrohydrodynamic, magnetic, magnetohydrodynamic, thermal, and acoustic actuations, to generate perturbation within the fluid streamlines to achieve higher mixing efficiencies [14,15,16,17,18,19]. Acoustic micromixers have attracted growing attention owing to their distinct advantages, such as being non-contact, less invasive, and free from dependence on fluid conductivity and permittivity. Generally, acoustic micromixers can be classified into bulk acoustic wave (BAW)-based micromixers and surface acoustic wave (SAW)-based micromixers.

In BAW-based micromixers, an electrically excited ultrasonic elastic wave transmits into the microchannel and generates pressure fluctuation, which creates empty voids in liquid. Bubbles can be induced by the empty voids in a liquid with an acoustic wave. This induction of bubbles can be characterized as acoustic cavitation [20,21]. The interaction between a bubble and acoustic pressure fluctuation causes bubble oscillation, which disturbs flow streams, resulting in mixing enhancement [18,22,23,24,25,26,27,28]. The initiation of inertial cavitation generally requires that pre-existing nuclei, e.g., stabilized gas pockets, be driven by ultrasound pressure waves with an amplitude exceeding a certain threshold value [20]. In the megahertz frequency range, the nucleation process typically requires pressure amplitudes sufficient to produce inertial cavitation, also called transient cavitation [20]. When an acoustic longitudinal wave propagates through a liquid, the total external pressure *P*(*t*) experienced by a macroscopic volume element is composed of two parts: a static part and a time-varying part [29]. Thus, *P*(*t*) is given by

(1) $$P\left(t\right)=P_{0}+P_{A}\sin\left(\omega t\right)$$

where *P*_0_ is the static pressure, usually the atmospheric pressure; *P*_A_ is the maximum amplitude of the acoustic pressure; and *ω* is the angular frequency of the acoustic wave. Given *P*_A_ > *P*_0_, the liquid will be in tension, as represented by negative external pressure, during part of the acoustic cycle. The tension produced by ultrasound is ultimately responsible for producing cavitation. When the tension exceeds the cavitation threshold for water, as required by the homogeneous nucleation theory, a new cavity will be produced in liquid. Introducing artificial cavitation nuclei in the form of ultrasound contrast agents (UCAs) has demonstrated some success in promoting ultrasonic cavitation [30]. Higher harmonics with frequencies beyond 10 MHz are strongly damped while passing through liquids. This energy deposition leads to a temperature increase [31]. The activation of pre-existing nuclei may either be thermally mediated, resulting from ultrasonic heating or mechanically driven, resulting from negative acoustic pressure [32]. The bubble dynamics have been studied by many researchers, and the additional heat can be generated along with ultrasonic cavitation. The work of Hilgenfeldt et al. [31] and ensuing investigations by Yang et al. [33] on the modeling of bubble dynamics reveal that two mechanisms can explain the additional heat generation: viscous damping from the radial motion of stable cavities and local absorption of the acoustic emissions from inertial bubble collapses.

SAW is an acoustic wave propagating along the surface of an elastic material within a depth that is approximately equal to its wavelength. The most important component of SAW-based devices is metal interdigital transducers (IDTs) deposited on the surface of a piezoelectric substrate. In recent years, SAW-based microfluidic technologies have gained important roles in biomedical applications and health care for their inimitable features such as high precision, high energy efficacy, no harm to biological objects, convenient system integration, and mass production [34,35,36,37,38,39,40]. The applicability of SAW in the alignment of blood cells in microchannels and particles in droplets has been demonstrated by our previous work [41,42]. SAW-based micromixers usually enhance mixing efficiency by varying the configuration of IDTs and microchannels [6,43,44,45,46]. Attention has been focused on the mixing efficiency a few seconds after the IDTs are excited in most of these proposed SAW-based micromixing demonstrations. Due to the viscous absorption of acoustic energy in liquid and in surrounding PDMS material, the liquid temperature would increase, making SAW a promising approach to microfluidic heating [47,48]. However, the regulation of temperature is a critical parameter in many physical, chemical, and biological applications [49]. It is necessary to maintain the viability of cells or tissues in many processes. Due to the high dependence of protein stability on temperature, a temperature increase of even a few degrees may trigger a heat shock response of proteins [50]. Although a tiny temperature increase would not disrupt the integrity of the cell membrane, the function and internal structure of the cell may have been severely affected, such as protein misfolding, entanglement and/or unspecific aggregation of proteins, cell cycle arrest, and cytoskeleton defects [51]. Moreover, temperature fluctuation is also detrimental to the performance of SAW microfluidic devices since the propagation velocity of acoustic waves is sensitive to temperature [52]. Therefore, the temperature increase is a problem that cannot be ignored for SAW microfluidic devices. Hilgenfeldt et al. [31] showed that for short-pulse insonation (similar to that employed in diagnostic ultrasound) in a blood-like medium, viscous damping had little effect on temperature increases (~10 K or less) near the bubble, while heat dissipation could result in temperature increases of up to 100 K near the bubble wall if the driving amplitude increased to the upper limit of the diagnostic range at ≈ 30 atm. Therefore, it is worth considering what happens in a microchannel in a longer time range, such as whether there is bubble generation or considerable temperature increases, especially when the mixing operation works in a continuous mode.

Here, we report bubble-enhanced mixing induced by standing surface acoustic waves (SSAWs) in a microchannel. An acoustofluidic microdevice has been fabricated by lift-off process and soft lithography. We experimentally demonstrated that a significant enhancement in the mixing efficiency could be achieved by generating bubbles in a microchannel with SSAWs. With an applied voltage of 5 V, 50 times amplified, the proposed mixing microdevice could achieve 90.8% mixing efficiency within 60 s at a flow rate of 240 μL/h. Our results also suggest that despite the promotion of acoustic cavitation, the temperature increase was harmful to microfluidic devices.

## 2. Materials and Methods

**Figure 1:**
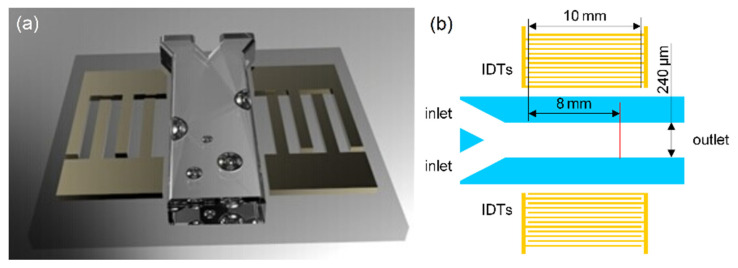
Schematic of the assembled SSAW mixing device: (**a**) 3D schematic and (**b**) 2D schematic. The acoustic aperture of IDTs is 10 mm. The main channel is 240 μm in width. The dimension ratios in (**a**,**b**) do not match the real ones. The propagating SSAW wavelength is defined by the IDTs’ finger width and spacing, both of which were designed as λ/4. Actuated by the SSAW, a mixing process can be expected.

**Figure 2:**
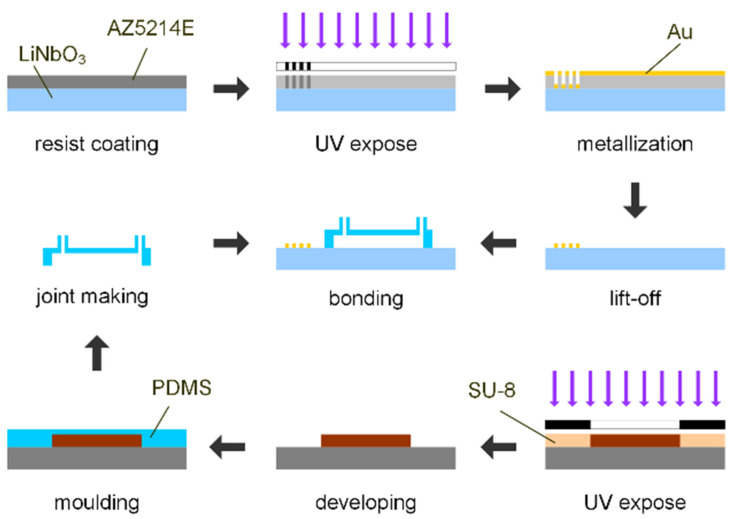
Fabrication process of the SSAW mixing device.

The proposed microdevice was composed of two sets of IDTs patterned on a piezoelectric substrate (128° Y-cut LiNbO_3_) and a Y-shaped polydimethylsiloxane (PDMS) microchannel layer bonded to the LiNbO_3_ substrate. Figure 1 is a schematic of the assembled microdevice. The two sets of IDTs, patterned by the lift-off process, are parallel to the Y-channel. The channel was fabricated using soft lithography and designed with two inlets and one outlet channel. The main channel between inlets and outlet was 240 μm in width and 100 μm in height. The straight red line in Figure 1b indicates the cross-sectional location chosen for the quantitative measurement of mixing efficiency. Figure 2 shows the fabrication process flow of the microdevices.

To manifest the mixing of two fluids inside the channel, deionized water (DI water) and DI water dyed with blue ink were injected into two inlets continuously. The flow rates of both inlets were set to 120 μL/h. An RF signal generator (E4422B, Agilent, Palo Alto, CA, USA) producing an AC signal at 2.8 MHz and a power amplifier (100A250A, Amplifier Research, PA, USA) amplifying signal were used. The signal amplified 50 times was equally partitioned into two separate signals and applied to the two sets of IDTs for SSAW generation. The assembled device was mounted onto the stage of a microscope (ECLIPSE LV100, NIKON, TOKYO, JAPAN) for visualization. The dye distribution in the microchannel was imaged and recorded using a CCD camera (DCRDVD803E, SONY, TOKYO, JAPAN). SSAW-based mixing experiments at different input voltages (3 V, 3.5 V, 4 V, 4.5 V, 5 V, 5.5 V, and 6 V) were conducted.

## 3. Results and Discussions

**Figure 3:**
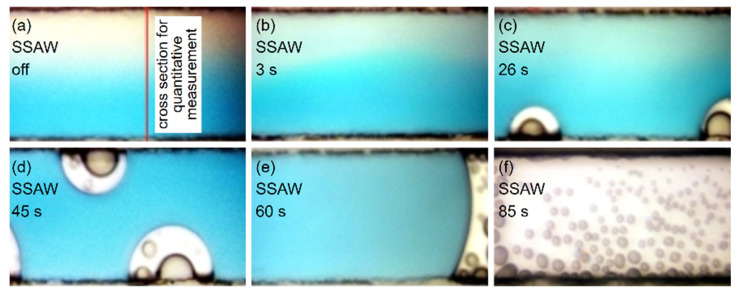
Top view of the channel in our SSAW-based mixing device (input voltage 6 V, 50× amplified): (**a**) SSAW off; (**b**) 3 s after SSAW was switched on; (**c**) 26 s after SSAW was switched on; (**d**) 45 s after SSAW was switched on; (**e**) 60 s SSAW after was switched on; (**f**) 85 s after SSAW was switched on.

**Figure 4:**
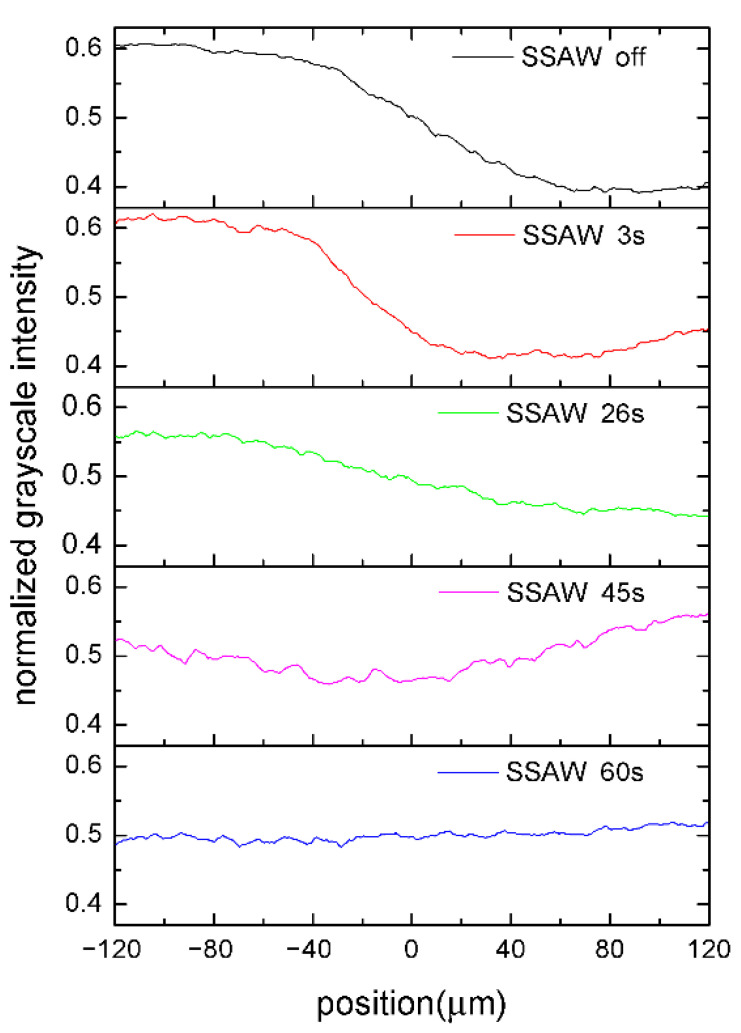
The normalized dye intensity distributions along channel width.

**Figure 5:**
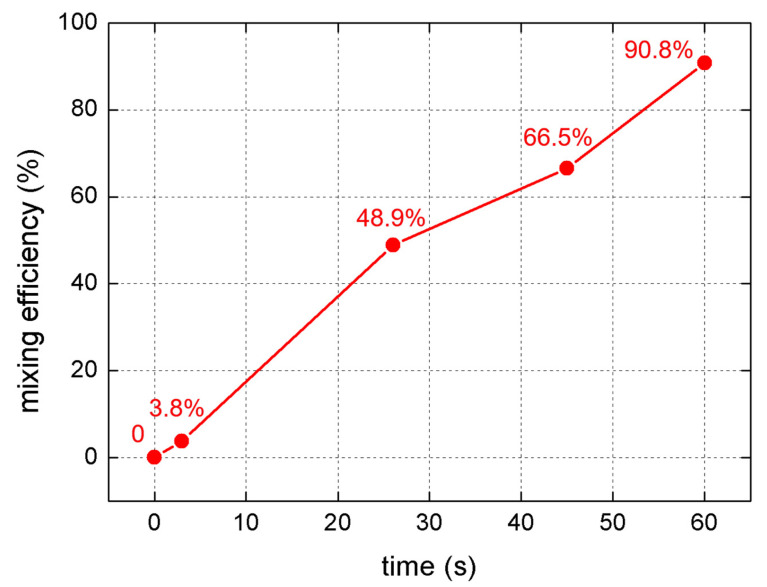
Variation of mixing efficiency.

Figure 3 shows the top view of the PDMS channel in our SSAW-based mixing device when the input voltage of the RF signal was set to 6 V and amplified 50 times. To provide a quantitative characterization of the mixing efficiency inside the microchannel, the recorded color images in Figure 3 were transformed into grayscale images. We chose a cross section located 8 mm downstream from the IDTs’ working area, as indicated by the straight red line in Figure 1b and Figure 3a. The normalized grayscale intensity distributions along such a cross section are displayed in Figure 4. In Figure 4, a negative value of position is used for the upper half of the channel width and a positive value is used for the lower half. Grayscale refers to the color depth of points in a grayscale image. The lighter the color, the higher the gray value. Furthermore, the mixing efficiency was calculated using the following expression [22]:

(2) $$\sigma =1-\frac{\sqrt{\frac{1}{N}\sum_{i=1}^{N}{\left(\bar{I_{i}}-\bar{I_{\infty }}\right)}^{2}}}{\sqrt{\frac{1}{N}\sum_{i=1}^{N}{\left(\bar{I_{0i}}-\bar{I_{\infty }}\right)}^{2}}}$$

where *N* represents the total number of pixel points examined along the chosen cross section, $\bar{I_{i}}$ is the normalized grayscale intensity at each pixel point, $\bar{I_{0i}}$ is the normalized grayscale intensity at each pixel point without SSAW actuation, and $\bar{I_{\infty }}$ is the normalized intensity in the complete mixing state. Figure 5 shows the variation in mixing efficiency at different moments after the SSAW is switched on.

When the IDTs were not actuated, the blue-dyed water was confined to the lower half flow in the channel by laminar streamlines, as shown in Figure 3a. The black normalized grayscale intensity curve could be regarded as composed of three segments, as shown in Figure 4. The left and right segments are almost horizontal lines, indicating that the water and blue-dyed water flowed separately. The middle segment is a slant, indicating that mixing occurred slowly via diffusion since the flow in the microchannel is laminar flow. When the SSAW was switched on, the blue area became wider while the color became faint in the middle of the channel, as shown in Figure 3b. The red curve in Figure 4 shows similar characteristics to the black curve. The slight difference between them is that the slant segment of the red curve moved leftwards compared with the black one. At 3 s after the SSAW was switched on, the mixing was still poor since the mixing efficiency was still below 5%, as shown in Figure 5. This suggests that the SSAW-induced vibration does not make a significant contribution to the mixing in our case. Twenty seconds after the SSAW was switched on, bubbles began to emerge and grow on the sidewall of the channel. Figure 3c shows two generated bubbles on the lower channel sidewall at 26 s after the SSAW was switched on. With the emergence of the bubble, the mixing was significantly enhanced, indicated by the mixing efficiency of 48.9% after 26 s of SSAW actuation. The blue curve in Figure 4 is close to a horizontal line with a normalized grayscale intensity of 0.5. The mixing efficiency reached 90.8% the moment 60 s after the SSAW was switched on. The increasing mixing efficiency shows a certain degree of synchronization with the generation of bubbles. Table 1 compares the main parameters between existing acoustic-based micromixers and the proposed mixer in this study. The proposed mixer achieves a level of mixing efficiency similar to the listed existing acoustic-based micromixers. Although our mixer takes a longer time to achieve effective mixing, 60 s is almost negligible when the mixing operation works in a continuous mode. To improve the time efficiency of mixing, the parameters of IDTs and the shape of the microchannel are two points of concern that are possible to optimize. Furthermore, the resonance frequency should be swept near the theoretically calculated resonance frequency for higher time-efficiency of mixing at lower input voltage.

The bubbles that emerged grew but then receded and disappeared before their diameters reached about one-third of the channel width until 40 s after the SSAW was switched on when newly generated bubbles began to keep growing gradually rather than receded and disappeared. Figure 3d shows two newly generated bubbles 45 s after the SSAW was switched on. As SSAW actuation went on, the bubbles grew and merged to form gas columns, dividing mixed liquid into columns and blocking liquid columns from flowing. The gas columns kept growing and extended in two directions along the channel, resulting in no liquid flow observed. Figure 3e shows the blocked fluid column resulting from the growing and merging of the bubbles. Figure 3f shows a bubble column.

**Figure 6:**
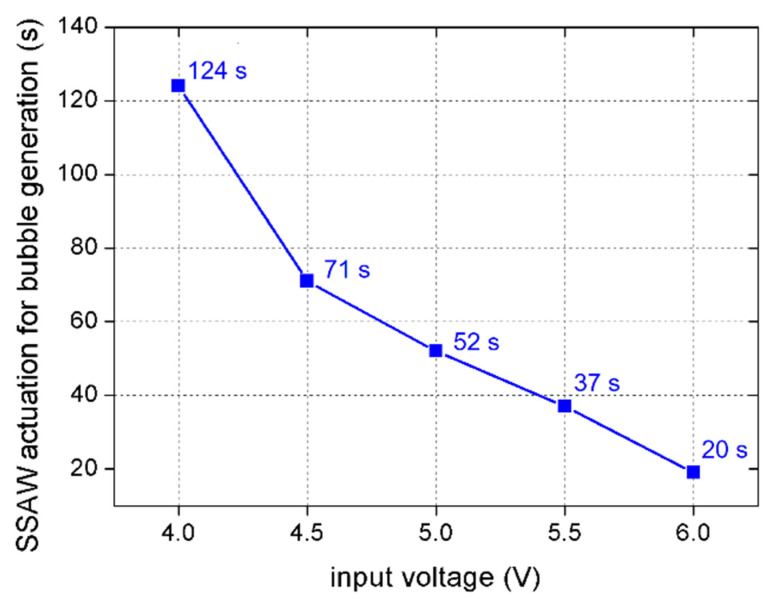
SSAW actuation time for generation of the first bubble at different input voltage.

When the input voltage was 3 V or 3.5 V, the energy of SSAWs was insufficient for the generation of bubbles within our observing time (300 s), and the mixing was incomplete. When the input voltage was 4 V or above, bubble generation in the microchannel was observed within 300 s. The actuation time of SSAWs for the first bubble generation decreased with input voltage. As shown in Figure 6, it took 124 s to generate the first bubble at 4 V, while it took only 20 s at 6 V.

There are particles in DI water, especially in DI water dyed with blue ink, since the blue ink used in our experiments includes soluble blue dye, gum arabic, and preservatives. The particles in ink and DI water make a good cavitation nuclei. That is why bubbles first emerged on the lower sidewall, as shown in Figure 3c. As the bubbles grew and disappeared constantly, the DI water dyed with blue ink mixed with the DI water in the microchannel. The particles migrating to the upper sidewall may have promoted ultrasonic cavitation in the upper sidewall. Then, the bubbles emerged on the two sidewalls, as shown in Figure 3d.

In addition to bubble generation and mixing enhancement, there was a perceptible temperature increase in the PDMS after the RF power was switched on. As shown in Figure 3c–e, there were nucleus-like objects with changing sizes inside the generated bubbles. Several seconds after the bubble generation, the nucleus-like objects emerged, grew, and merged once they came into contact. They merged into a liquid column when they contacted the liquid–bubble interface. This suggests that nucleus-like objects appear to be droplets depositing on the inner surface of the microchannel. Unfortunately, at an input voltage of 6 V, dozens of seconds after the merged bubbles blocked the water flow in the microchannel, the PDMS layer was peeled off from the LN substrate. The flow rates of both inlets were set to 120 μL/h. Once a gas column blocks the liquid flow, the pressure inside the microchannel keeps growing until it reaches the bonding strength limit. The temperature increase accelerates the increase0 in pressure. The bonding between the PDMS and LN substrates is destroyed. That is why the PDMS layer is peeled off from the substrate.

The temperature increase may promote the growth of a nuclei by raising the vapor pressure or the partial pressure of dissolved gas in a liquid. When the RF power increases, the acoustic-induced heating increases; therefore, more bubble nuclei are recruited, and bubbles are driven into more violent oscillation. In our experiments, it takes a shorter time for bubble generation at a higher input voltage of RF power, not just because higher voltage actuates higher maximum amplitude of the acoustic pressure but because it takes a shorter time to heat the liquid and the surrounding PDMS material to promote ultrasonic cavitation. Our experimental results show that the microchannel of the SAW device can be heated to a high temperature. At an input voltage of 6 V, our PDMS microchannel is destroyed by acoustic-induced high temperatures.

Despite the unfortunate destruction of our SSAWs-based mixing microdevice, our work provides a demonstration of mixing enhancement by SSAW. In further research, the configuration of IDTs and microchannels should be optimized to improve time efficiency and to reduce temperature rise. Furthermore, the resonance frequency should be swept near the theoretically calculated resonance frequency for higher time-efficiency of mixing at lower input voltage. If RF power is controlled in a reasonable range, which is enough for steady oscillation of bubbles but induces less temperature rise, SAW actuation is a quite promising approach in micromixing application.

## 4. Conclusions

BAW-based micromixers usually achieve mixing enhancement with acoustic-induced bubbles, while SAW-based micromixers usually enhance mixing efficiency by varying the configuration of IDTs and microchannels. In this paper, we proposed bubble-enhanced acoustic mixing induced by SSAWs in a microfluidic channel. An acoustofluidic microdevice, composed of two sets of IDTs patterned on an LN substrate and a Y-shaped PDMS microchannel bonded to an LN substrate, was fabricated by a lift-off process and soft lithography. The micromixing between DI water and DI water dyed with blue ink was experimentally investigated. We demonstrated that a significant enhancement in the mixing efficiency could be achieved by generating bubbles in a microchannel with SSAWs. With the applied voltage of 5 V, 50 times amplified, the proposed mixing microdevice could achieve 90.8% mixing efficiency within 60 s at the flow rate of 240 μL/h. The bubbles were generated from acoustic cavitation. The temperature rise resulting from viscous absorption of acoustic energy also assisted the acoustic cavitation. Damage to microchannel resulting from SSAWS-induced temperature increase was observed at an input voltage of 6 V 50 × amplified, which suggests that a temperature increase is harmful to microfluidic devices, despite the promotion of acoustic cavitation. To achieve stable oscillation of the bubbles, to improve the time efficiency, and to control the temperature rise, further investigation is necessary. For the wide and safe application of SAW in microfluidic systems, more attention should be paid to temperature monitoring and regulation in SAW-based microdevices, especially in chemical, biological, and biomedical applications. 

## Figures and Tables

**Table 1:** Comparison of main parameters with other research.

Author	Actuation	Frequency	Amplification	Input Voltage	Flowrate	Mixing Efficiency	Mixing Time
Wang et al. [22]	BAW	1.5 kHz	20×	5V_pp_ ^*^	5 mL/h	<90%	1–2 s
Nam et al. [44]	FSAW	9.2 MHz		21V_pp_	100 mL/min	>90%	
Ahmed et al. [46]	SAW	140 MHz		12V_pp_	200 μL/min	>90%	
Our group	SSAW	2.8 MHz	50×	6V_pp_	240 μL/h	90.8%	60 s

^*^ Peak-to-peak voltage.

## Data Availability

Not applicable.
